# Fine mapping of the uterine leiomyoma locus on 1q43 close to a lncRNA in the *RGS7-FH* interval

**DOI:** 10.1530/ERC-15-0208

**Published:** 2015-08

**Authors:** Brahim Aissani, Kui Zhang, Arjen R Mensenkamp, Fred H Menko, Howard W Wiener

**Affiliations:** 1 Department of Epidemiology, R217J, School of Public Health, University of Alabama at Birmingham, 1665 University Boulevard, Birmingham, Alabama, 35294-0022, USA; 2 Department of Biostatistics, School of Public Health, University of Alabama at Birmingham, 1665 University Boulevard, Birmingham, Alabama, 35294-0022, USA; 3 Department of Human, Genetics Radboud University Medical Center Nijmegen, Nijmegen, The Netherlands; 4 Netherlands Cancer Institute, Amsterdam, The Netherlands

**Keywords:** HLRCC, uterine fibroids, FH, fumarate hydratase, RGS7, PLD5

## Abstract

Mutations in fumarate hydratase (*FH*) on chromosome 1q43 cause a rare cancer syndrome, hereditary leiomyomatosis and renal cell cancer (HLRCC), but are rare in nonsyndromic and common uterine leiomyoma (UL) or fibroids. Studies suggested that variants in *FH* or in a linked gene may also predispose to UL. We re-sequenced 2.3 Mb of DNA spanning *FH* in 96 UL cases and controls from the multiethnic NIEHS-uterine fibroid study, and in 18 HLRCC-associated UL probands from European families then selected 221 informative SNPs for follow-up genotyping. We report promising susceptibility associations with UL peaking at rs78220092 (*P*=7.0×10^−5^) in the *RGS7-FH* interval in African Americans. In race-combined analyses and in meta-analyses (*n*=916), we identified promising associations with risk peaking upstream of a non-protein coding RNA (lncRNA) locus located in the *RGS7-FH* interval closer to *RGS7*, and associations with tumor size peaking in the distal phospholipase D family, member 5 (*PLD5*) gene at rs2654879 (*P*=1.7×10^−4^). We corroborated previously reported *FH* mutations in nine out of the 18 HLRCC-associated UL cases and identified two missense mutations in *FH* in only two nonsyndromic UL cases and one control. Our fine association mapping and integration of existing gene profiling data showing upregulated expression of the lncRNA and downregulation of *PLD5* in fibroids, as compared to matched myometrium, suggest a potential role of this genomic region in UL pathogenesis. While the identified variations at 1q43 represent a potential risk locus for UL, future replication analyses are required to substantiate our observation.

## Introduction

Uterine leiomyoma (UL) are benign neoplasms that arise from the smooth muscle cells of the uterus. Despite their benign nature, UL are responsible for significant gynecologic morbidities including excessive bleeding, pelvic pain, urinary incontinence, infertility, and pregnancy complications (Stewart 2001, Walker & Stewart 2005). As a consequence of this morbidity, uterine fibroids are the primary indication for hysterectomy, with an incidence rate of hysterectomies of 5.5 per 1000 women in the United States (Farquhar & Steiner 2002) and accounting for about $9.4 billion of public health burden. Cumulative exposure to estrogen is believed to be a major etiologic factor (Andersen 1996) and factors that may influence the hormonal milieu, such as obesity, are also believed to be associated with risk (Schwartz *et al*. 2000). Clearly established risk factors are age (increasing risk with increasing premenopausal age), menopause (risk decreases with menopause) and African American (AA) ethnicity (higher risk compared with that of non-Hispanic Whites) (Baird *et al*. 2003).

Several lines of evidence for a genetic basis of UL have been demonstrated in familial aggregation and twin studies (Kurbanova *et al*. 1989, Vikhlyaeva *et al*. 1995, Luoto *et al*. 2000, Sato *et al*. 2002, Van Voorhis *et al*. 2002). Candidate susceptibility genes have emerged from genome-wide association studies (GWAS) (Cha *et al*. 2011, Eggert *et al*. 2012), mapping by admixture linkage disequilibrium (MALD) studies in AAs of signals for UL (Wise *et al*. 2012, Zhang *et al*. 2015) and from our candidate gene approach implicating genes encoding components of the extracellular matrix (Aissani *et al*. 2015). Studies conducted in Biorepository at Vanderbilt University study (BioVu) and right from the start study (RFTS) reported replications of GWAS findings in the Japanese population (Cha *et al*. 2011) of candidate trinucleotide repeat containing 6B (*TNRC6B*) and blocked early in transport 1 homolog (*BET1L*) genes (Edwards *et al*. 2013*a*,*b*). Exome sequencing in 18 fibroids and matched normal myometria has implicated the gene encoding the mediator complex subunit 12 (*MED12*) in UL (Makinen *et al*. 2011). Further examinations of whole genome sequences and gene-expression profiling in a set of 38 fibroids and matched myometria led to the hypothesis that a chromothripsis-like event drives the pathogenesis of UL leading to translocations of *HMGA2* and *RAD51B*, and to other chromosomal aberrations including the collagen *COL4A5-COL4A6* locus (Mehine *et al*. 2013). In contrast to these somatic mutations, germline mutations associated with UL were observed in the gene encoding the Krebs cycle enzyme fumarate hydratase (*FH)* in hereditary leiomyomatosis and renal cell carcinoma (HLRCC; OMIM 150800), a rare and dominantly-transmitted Mendelian syndrome (Alam *et al*. 2001, Launonen *et al*. 2001, Tomlinson *et al*. 2002) as well as in rare cases of nonsyndromic UL (Barker *et al*. 2002, Kiuru *et al*. 2002). Furthermore, there was no evidence for epigenetic inactivation of *FH* in UL and leiomyosarcoma, the malignant counterpart of UL (Barker *et al*. 2006). Hereafter, we will use ‘UL’ to refer to nonsyndromic fibroids (common form) and ‘HLRCC’ to syndromic fibroids (familial and rare form).

Our initial examination of an extended chromosome 1q43 region spanning *FH* and other suspected susceptibility loci highlighted multiple signals for association with risk and size of UL in NIEHS-UFS (Aissani *et al*. 2013). However, we could not assess whether regulator of G-protein signaling 7 (*RGS7*), *FH* or any of the uncharacterized gene loci located between them was the true target in UL because the association with UL peaked in the *RGS7-FH* interval. The aim of the present study was to: i) refine the location of the candidate gene(s) for risk and size of UL; ii) test whether *FH* is also mutated in UL but a presumably marked allelic heterogeneity at this locus, similar to that observed in HLRCC (Tomlinson *et al*. 2002, Bayley *et al*. 2008) precluded detection of associations; and iii) test for the presence of alternative susceptibility loci in the *FH* region that might associate with UL and would explain up to 40% of women with HLRCC-associated UL that do not carry mutations in *FH* (Tomlinson *et al*. 2002).

To this end, we re-sequenced 2.3 Mb across *FH* in a subset of NIEHS-UFS UL cases and controls (*n*=96) and in 18 probands from Dutch families segregating HLRCC, and identified candidate single nucleotide polymorphisms (SNPs) for follow-up genotyping in the remaining NIEHS-UFS sample (*n*=820). We report new association data in NIEHS-UFS implicating a large intergenic non-coding RNA (lnc-RNA) located between *RGS7* and *FH* in nonsyndromic UL. We confirm previously reported *FH* mutations in nine of the 18 re-sequenced HLRCC probands (Smit *et al*. 2011) and report *FH* mutations in only two UL cases and also in one UL-free control.

## Material and methods

### Study population

Detailed characteristics of the study population have been reported (Baird *et al*. 2003, Aissani *et al*. 2013). Briefly, a random sample of women, aged 35–51 years, was selected from a computerized list of members of a prepaid urban health plan for enrollment in the NIEHS-UFS (Baird *et al*. 2003). Of the enrolled premenopausal women, 1045 (93%) had ultrasound examinations and available DNA specimens self-identified as having an AA (*n*=574), non-Hispanic European American (EA, *n*=394) or other (*n*=77) ethnic background. The NIEHS-UFS and the present sub-study were approved by the Human Subjects Review Boards at the NIEHS, George Washington University and University of Alabama at Birmingham respectively. Participants gave written informed consent in accordance with these Review Boards.

### Ascertainment

Fibroid status was assessed by ultrasound screening at baseline or by medical record review in about 84 and 90% of the AA and EA participants respectively. For women who had a pelvic ultrasound examination recently at a health plan (24.7% in AA and 12.1% in EA), the radiology records from that examination were used to assess fibroid status. The remaining premenopausal participants (59.5% in AA and 76.8% in EA) were asked to have a pelvic ultrasound examination at a primary care site. Women for whom neither ultrasound nor medical record review could be conducted were excluded. Both a transabdominal and a transvaginal ultrasound examination were performed. The abdominal portion evaluated fibroid change arising from the upper uterus that would not be readily seen with the transvaginal approach alone. Tumor size was classified in three categories of size (small, medium and large) measured by the diameter of the tumors (S≤2 cm, 2<M<4 cm, L≥4 cm). For participants diagnosed with multiple tumors, the largest tumor determined the size category.

### Covariates

The covariates included age, age at menarche, parity after age 25 (earlier births were not significantly related to fibroid development in the NIEHS-UFS) (Baird *et al*. 2003), BMI and physical activity.

### Family recruitment

Probands and family members of HLRCC families visited outpatient clinics throughout The Netherlands. DNA was sent to the Genome Diagnostics Laboratory at the Radboud University Medical Center in Nijmegen (The Netherlands). *FH* gene analysis was performed as previously described (Smit *et al*. 2011). All patients approved the anonymous use of their DNA, in accordance with Dutch law.

### Genotyping and sequencing

#### DNA preparation

DNA was extracted from blood samples using the QIAAMP DNA Mini Kit (Qiagen) procedure and isolated DNA has undergone purification prior to quantification by the PicoGreen assay (Invitrogen).

#### Next-generation sequencing

Having observed several peaks of association with risk and tumor size in the initial study (Aissani *et al*. 2013), we opted for a two-stage approach (re-sequencing and follow-up genotyping) to fully investigate DNA sequence variation in the target genomic region. Illumina HiSeq2000 sequencing system (Illumina, Inc., San Diego, CA, USA) was used to re-sequence about 2.3 Mb of DNA (from downstream of *RGS7* to upstream of phospholipase D family, member 5 (*PLD5*) spanning the *FH* locus in a subset of 96 NIEHS-UFS samples representative of each of the affection status (UL cases or controls), BMI categories and ethnic groups (EA and AA). Eighteen probands from Dutch families with confirmed or suspected HLRCC and two female relatives with no HLRCC-associated UL were also selected for re-sequencing.

#### Genotyping

We selected a set of 264 SNPs for follow-up genotyping consisting of 45 custom assays (new SNPs identified through re-sequencing) and 219 validated SNP assays from dbSNP. The selection was based on different criteria including but not limited to the statistical significance (analysis of combined AA and EA cases and controls), minor allele frequency (MAF>5%), SNP location in gene functional regions (for intragenic SNPs, further selection was based on linkage disequilibrium with associated SNPs in the initial study (Aissani *et al*. 2013)) and SNP assay designability. We used the Illumina GoldenGate platform (Illumina, Inc.) to genotype 820 NIEHS-UFS (477 AA and 343 EA) samples. Reliability in genotyping data was assessed by inclusion of blind duplicates (two duplicates per 96-well plates) and HapMap positive control samples (four controls per 96-well plates) as required by the Genetic Resources Core Facility of the Johns Hopkins University.

### Statistical analysis

#### Quality control

A call rate of 95% and a concordance rate of 100% between duplicates were assigned as quality control thresholds of the genotyping data. Prior to their inclusion in the analysis, SNP calls were examined separately in each ethnic group and affection status for adherence to Hardy–Weinberg equilibrium (HWE) using the Pearson's *χ*^2^ test and SNPs showing significant deviation (*P*<0.01) from HWE in the controls were excluded.

#### Association testing

Model-free Discriminant Analysis of Principal Components (DAPC) (Jombart *et al*. 2010) based on a total set of 4363 SNPs from over the genome was used in a previous study to defined clusters of genetically related individuals in NIEHS-UFS (Aissani *et al*. 2013). Logistic regression models adjusted for covariates were fitted to the data to evaluate the association between SNP genotypes and the risk for UL modeled as a dichotomous outcome (case and control design) or as polytomous outcome in either case-only design (three-level outcome) comparing tumor size categories small (S), medium (M) and Large (L) (S vs M, L; S, M vs L) or in a design that also included controls (four-level outcome) with no tumor (N) (N vs S, M, L; N, S vs M, L; N, S, M vs L). For the polytomous outcome, *P* value is reported only for the SNPs that met the assumption of proportional odds. In logistic regression modeling, the most frequent homozygous genotype in the controls (or category with the lowest level in proportional odds models) served as the reference genotype. The likelihood ratio test provided estimates of the statistical significance for each univariate SNP test as two-sided *P* values. Bonferroni correction was used to adjust for multiple testing and *P* values of less than the threshold (0.05/number of tested SNPs) in race-stratified analyses or in pooled analyses were deemed to be statistically significant. In pooled analyses, the logistic regression models were further adjusted for the SNP by race interaction term. Meta-analyses were conducted using random-effect variance and DerSimonian-Laird estimator (DerSimonian & Laird 1986) in the Metafor package (Viechtbauer 2010).

In contrast to HLRCC, which is inherited in an autosomal-dominant mode, there is no *a priori* knowledge on the genetic model underlying UL. Therefore, analyses were conducted under dominant and additive genetic models as well as in genotypic tests (model-free 2-d.f. test) but only data from additive models are shown.

#### Gene expression analysis

We used publicly available expression data from a study of uterine fibroids (Guo *et al*. 2014) to evaluate the functional impact of identified candidate loci. We retrieved raw expression data for lncRNA and *cis* mRNAs in uterine fibroids and matched myometrium from the EMBL-EBI Array Express website (http://www.ebi.ac.uk/arrayexpress/experiments/E-GEOD-52618/files/). The raw data contained expression of 62 738 probes from five groups of matched samples (five myometrium, five small fibroids and five large fibroids). Log2 transformation followed by quantile normalization (Bolstad *et al*. 2003) were used to normalize the raw data. The three probes corresponding to the candidate lncRNA, *FH*, and *RGS7* were identified through BLAST and their normalized expression levels were used for the calculation of Pearson's and Spearman's correlation coefficients with R (http://www.r-project.org/) for co-regulated lncRNA-mRNA expressions.

## Results

### Quality control

A subset of 43 (16%) SNPs consisting both of custom and validated SNP assays have failed genotyping. Among the remaining SNPs, the call rate was 99.7% and the concordance rate among duplicates was 100%. A possible explanation to these genotyping failures is the proximity among SNPs, which can hinder the multiplex GoldenGate assay even by observing the minimal inter-SNP distance of 60 bp recommended by the manufacturer.

### Next-generation sequencing and *FH* mutation analysis

A minimum sequencing depth of 50× was achieved and re-sequencing failed only for a single NIEHS-UFS sample. A total of 21 078 SNPs (on average 1 SNP/109 bp) including a subset of 253 gene regulatory and coding SNPs was identified. The higher polymorphism frequency compared to the 1000 genome project can be explained by the higher coverage depth in our next-generation sequencing (NGS) assay (50× vs 4×). We observed a total of 154 SNPs in the 22 kb-long *FH* gene (21 variants in coding and regulatory regions) in 113 quality control samples (93 NIEHS-UFS and 20 HLRCC samples) and confirmed the presence of *FH* mutations (Table 1) in nine of the 18 tested HLRCC probands as previously reported (Smit *et al*. 2011). The proband who carried *FH* mutation c.952C>T (p.His318Tyr) presented cutaneous leiomyoma and no UL.

In nonsyndromic UL, two missense mutations in *FH* exon 1 (c.53C>T and c.55G>T) were found in two UL cases and one control of AA descent (Table 1). Mutation c.55G>T (p.Ala19Ser) occurred in a single case out of 24 re-sequenced UL cases of AA descent (MAF=0.021 among AA cases and a MAF=0.0054 in the entire re-sequenced AA and EA case and control samples). Mutation c.53C>T (p.Pro18Leu) occurred in 2% of the AA cases and 1.1% in the entire re-sequenced sample.

### Follow-up association mapping in race-stratified analysis

Association of 221 post-NGS and QC-filtered SNPs with the risk of UL was evaluated in additive models separately in the AA and EA groups (Supplementary Figure S1, see section on supplementary data given at the end of this article). The results showed an association (*P*=6.5×10^−4^) at the intronic variant rs35914368 in the 5′ end of *RGS7* and statistically significant association (*P*=7.0×10^−5^) at rs78220092 in the *RGS7-FH* intergenic region in the AA group. The most significantly associated rs78220092 SNP has a MAF of about 0.14 in the AA group and is rare (<1%) in EA.

In proportional odds models, the associations with tumor size were marginal (Supplementary Figure S2) and reached the highest statistical significance at rs28627534 (*P*=0.0021), a common SNP located 3 kb downstream of microtubule-associated protein 1 light chain 3 gamma (*MAP1LC3*). We noticed that about 10% of the SNPs in AA and 50% EA did not meet the assumption of proportional odds. Close examinations of these SNPs showed that many had low MAF or were not polymorphic in EA.

### Follow-up association mapping in race-combined analysis and meta-analysis

**Figure 1:**
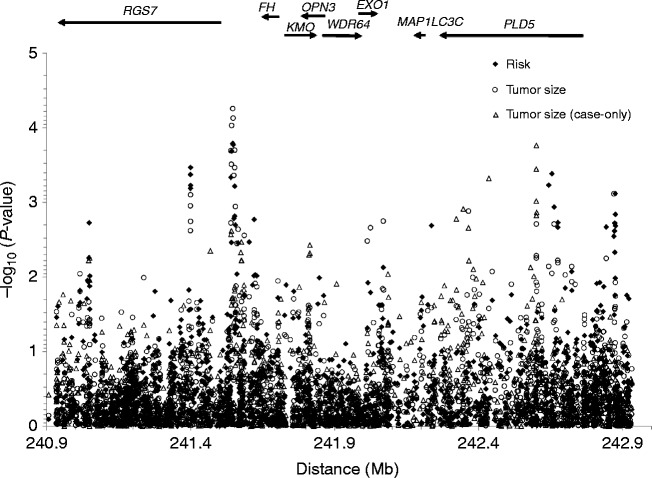
Fine mapping of chromosome 1q43 variants associated with risk and size of uterine fibroids in the NIEHS uterine fibroid study. The plot shows the strength of association (expressed as minus log10 of *P* value) between 1780 quality control-filtered single nucleotide polymorphisms (SNPs) and uterine fibroid outcomes (filled diamonds: risk; empty circles: tumor size including controls as the category with the lowest level; empty triangles: tumor size in case-only design) obtained for a pooled sample (525 African American and 391 European American individuals) using logistic regression models with adjustments for covariates (age, age-at-menarche, parity, BMI and physical activity) and for SNP by race interaction term.

To finely map the putative UL susceptibility locus on chromosome 1q43, we re-evaluated the association with the entire set of 1780 SNPs (1559 SNPs from the initial study (Aissani *et al*. 2013) that was performed only in race-stratified models and the 221 post-NGS SNPs) in analyses of pooled AA and EA samples and in meta-analyses. The small sample (*n*=70) representing the ethnic group defined as ‘other’ was excluded to allow the results of race-stratified and race-pooled designs to be compared. The results of the combined analysis (Fig. 1) and meta-analysis (Supplementary Figure S3, see section on supplementary data given at the end of this article) showed a prominent peak of association with risk centered in the intergenic interval delimited by centromeric *RGS7* and telomeric *FH* genes. More precisely, the association with risk peaked at rs2341938 (*P*=1.6×10^−4^) in the combined analysis and at the nearby rs78220092 SNP (*P*=5.4×10^−5^) in meta-analysis (Table 2 and Supplementary Table S1). The negative association with rs78220092 in the pooled sample is most likely driven by the low allele frequency of rs78220092 in the EA group.

### Gene annotation and functional correlates across the candidate *RGS7-FH* region

**Figure 2:**
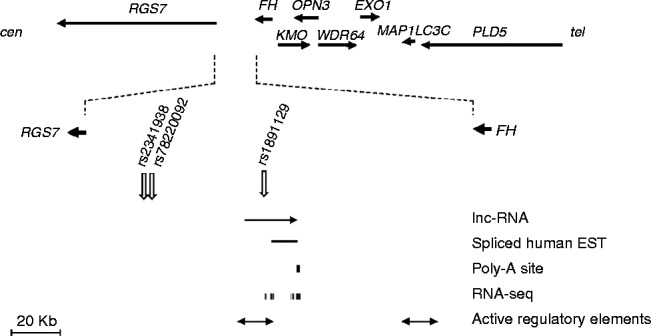
Genomic annotation of the candidate uterine leiomyoma locus on human chromosome 1q43. The genomic map shows the location of the single nucleotide polymorphism (SNP) sites at which the association with risk of uterine leiomyoma (UL) peaked in the pooled analysis (rs2341938) and meta-analysis (rs78220092) between centromeric (cen) regulator of G-protein signaling 7 (RGS7) and telomeric (tel) fumarate hydratase (FH). The map shows also the relative location of lnc-RNA TCONS_l2_00000923 containing the SNP (rs1891129) associated with UL and with FH expression in peripheral blood mononuclear cells.

No reference gene sequence maps to this genomic interval in the human genome assembly 19 (GRCh37/hg19). However, expressed sequence annotations from different sources indicate the presence of a large intergenic non-coding RNA (lncRNA) gene located about 30 kb telomeric to the peak of association (Fig. 2). Several SNPs located in the lncRNA locus showed moderate associations (*P*≤0.01) with either risk or tumor size (Supplementary Table S1). In particular, a common SNP (rs1891129 C>T at position 241 586 687) in the lncRNA that showed moderate associations with risk (*P*=0.017) and tumor size (*P*=0.037 in case only-design and *P*=0.0027 in the four-level design) (Table 2) is an expression quantitative trait locus (eQTL) significantly associated (*β* coefficient=23.9, *P*=0.003) with *FH* but not with *RGS7* (*β*=−2.26, *P*=0.31) expression (Supplementary Figures S4 and S5, see section on supplementary data given at the end of this article) in blood (Heinzen *et al*. 2008). No eQTL information was available in the SNPexpress database for the other candidate SNPs listed in Table 2 except for rs4660080, which showed no significant association with either *RGS7* or *FH*, and for the distal SNPs rs2654879 and rs6429360, which were not associated with *PLD5* expression.

Using published expression data (Guo *et al*. 2014), we tested whether regulation (up- or down-regulation) of the target lncRNA expression occurs in fibroids compared to matched myometrium. The results showed a threefold increase (Log2 fold change=1.62) in the lnRNA expression in the large fibroids as compared to the myometrium (*P*=0.02) (Supplementary Table S2). A marginal difference (*P*=0.09) in the lncRNA expression was observed between large and small tumors and no significant or marginal difference was observed between small tumors and myometrium, suggesting possible effects of this lncRNA on tumor size.

Compared to *UC.10*, a well-studied lncRNA overlapping ADAM metallopeptidase domain 12 (*ADMA12*) and reported to be significantly upregulated (*P*=5.2×10^−5^) in uterine fibroids, and to some extent to the target lncRNA in the present study, no significant change in gene expression was seen for the putative *cis*-regulated *FH* and *RGS7* genes.

Furthermore, we tested whether the lncRNA and the *cis* mRNA expressions were co-regulated and observed a moderate correlation in a global test (all tissues considered) for the lncRNA-*RGS7* pair (Pearson's *r*=0.52 and Spearman's *ρ*=0.58) and for *CU.10-ADAM12* (*r*=0.61 and *ρ*=0.51) but not for lncRNA-*FH* (Supplementary Table S3, see section on supplementary data given at the end of this article). Higher correlations were further seen for the lncRNA-*RGS7* pair when the analysis was restricted to the small and large fibroids (*r*=0.58 and *ρ*=0.71).

### Association of chromosome 1q43 SNPs with tumor size

The association with tumor size peaked at the risk-associated SNP rs2341938 (*P*=5.5×10^−5^) when a four-level response variable (no tumor, small, medium and large) was modeled (Fig. 1). In a case-only design (small, medium and large), the strongest associations was observed in intron 2 of *PLD5* at rs2654879 (*P*=1.7×10^−4^) in the combined analysis (Table 2 and Fig. 1). In meta-analysis, the association peaked at the low-frequency and intronic SNP rs316912 and extended to the proximal SNP rs28627534 (*P*=1.8×10^−3^) in the autophagy *MAP1LC3C* gene and to the distal SNP rs6429360 (*P*=1.8×10^−3^) in *PLD5*.

In the reported gene profiling study (Guo *et al*. 2014), *PLD5* ranked among the 25 genes with most dysregulated expression in fibroids. *PLD5* expression in large fibroids was 0.49-fold (down-regulation) that of the small fibroids (*P*=0.0076) and 0.41-fold that of the matched myometrium (*P*=0.003) (Supplementary Table S4, see section on supplementary data given at the end of this article). No significant change in *PLD5* gene expression was, however, observed between small fibroids and matched myometrium (*P*=0.409).

## Discussion

The aim of the current study was to refine the location of a susceptibility locus for UL in a suspected region of chromosome 1q43 containing *FH*, a gene mutated in HLRCC-associated UL but rarely in the common form of UL. Following-up to our initial study that pointed to putative susceptibility loci for risk and size of UL on chromosome 1q43, we conducted a two-stage re-sequencing and follow-up genotyping study and evaluated the predictive value of a stringent selection of 221 SNPs. We identified promising susceptibility associations (Bonferroni-corrected *P*=0.015 in the AA group) with the risk of UL located in the genomic region flanked by *RGS7* and *FH*. We also reported a promising association of tumor size (Bonferroni-corrected *P*=0.037), with the distal *PLD5* gene in the AA group. These results were supported in analyses of pooled EA and AA samples and in meta-analyses that also included SNPs typed in the initial study but previously tested only in race-stratified models (Aissani *et al*. 2013). Due to allele frequency heterogeneity among the two study populations, the associations were observed at different SNP sites but within a common region of association. While the identified variations at 1q43 represent a potential risk locus for UL, future replication studies are required to substantiate our observation.

We observed two missense mutations in *FH* exon 1, c.55G>T (p.Ala19Ser) and c.53C>T (p.Pro18Leu), that have not been reported in the TCA cycle gene mutation database (LOVD v.2. 0 Build 36) (Bayley *et al*. 2008). The Exome Aggregate Consortium (ExAC) database reported the p.Pro18Leu mutation with a frequency of 2.4% in Africans and of 0.02% in non-Finnish Europeans. p.Ala19Ser is not reported in ExAC but a similar mutation Ala>Ser is reported at the next codon 20 with a worldwide frequency of about 0.007%. The impact of these mutations on the pathogenesis of nonsyndromic UL is yet to be demonstrated. One cannot exclude the possibility that these three heterozygous *FH* mutations rather evoke HLRCC-associated UL than nonsyndromic UL. The strict occurrence of these two mutations in single AA cases makes difficult the interpretation of these results. To our knowledge only rare instances of HLRCC have been reported in the AA population (Wei *et al*. 2006), possibly because HLRCC was essentially studied in populations of European descent. Nonetheless, with two out of the 24 tested UL cases of AA descent carried heterozygous *FH* mutations, albeit with unknown pathogenicity, screening for *FH* mutations in the entire NIEHS-UFS sample is worth an undertaking to unequivocally assess the spectrum of *FH* mutations in nonsyndromic UL and to evaluate the potential contribution of HLRCC to the UL pool in NIEHS-UFS.

Re-sequencing of the candidate genomic region in 18 probands from European families with suspected or confirmed HLRCC confirmed the presence of *FH* mutations in nine of them as previously reported (Smit *et al*. 2011).

Our rationale for evaluating the relevance of the *FH*-linked region to the development of UL was motivated by early linkage studies suggesting the presence in this genomic region of susceptibility loci for UL (Gross *et al*. 2004), predisposing for prostate cancer (PCaP; OMIM 602759) (Berthon *et al*. 1998) and for factors affecting the risk of UL such as adiposity (Aissani *et al*. 2006) and serum level of sex hormone-binding globulin (SHBG) (Ukkola *et al*. 2002) (Supplementary Figure S6, see section on supplementary data given at the end of this article). Furthermore, a large-scale meta-analysis of GWAS also implicated this genomic region in age-at-menopause (Stolk *et al*. 2012), a known risk for UL (increasing risk with increasing age-at-menopause). Collectively, these and other independent observations (Aissani *et al*. 2013) led to our working hypothesis that an alternative susceptibility locus lies in the vicinity of *FH* and acts alone or in interaction with *FH* to increase the risk of UL in susceptible individuals (Aissani *et al*. 2013).

Fine mapping of the association signal near a lncRNA locus is consistent with our hypothesis for the implication of an alternative 1q43 gene in nonsyndromic UL because genetic variants influencing the transcription, sequence or structure of the lncRNA may interfere with the expression of the target *cis* gene. The sole gene profiling study to date that examined the expression of lncRNAs in fibroids provided the first functional evidence for the potential implication of this genomic region in UL. While the data of the gene profiling study are supportive of our fine mapping of the putative UL susceptibility locus to the *RGS7*-*FH* interval, they are not consistent with *FH* expression being regulated by the lncRNA.

The possibility that the association signal upstream of the target lncRNA overlaps a regulatory region with *cis* effects on the lncRNA expression and the target *cis* mRNA cannot be excluded. Several examples of co-regulated lncRNA and protein-coding loci by the same *cis*-rSNP, the so-called enhancer RNA (eRNA), have been reported (Almlof *et al*. 2014). The role of these eRNAs in mediating the function of the enhancer in directing basal gene expression was demonstrated in a recent study for the distal enhancer of the gonadotropin hormone α-subunit gene (Pnueli *et al*. 2015).

Collectively, the results of the eQTL study in blood from SNPexpress (Heinzen *et al*. 2008), the lncRNA and *cis*-coding gene expression study in fibroids and matched myometrium (Guo *et al*. 2014) and the present study converge on the hypothesis that the lncRNA is a potential target in the pathogenesis of non-syndromic UL; however, the first available lncRNA expression data appear to be more supportive of *RGS7* than *FH* as the target *cis* gene. Independent studies of co-regulated lncRNA and *cis* mRNA expressions in the RGS7-FH interval, as well as down-regulation of *PLD*5, in fibroids are needed to substantiate our observations.

Differential expression of non-coding RNA species and their target genes have been shown to associate with the risk of UL (Luo & Chegini 2008, Marsh *et al*. 2008, Zavadil *et al*. 2010, Georgieva *et al*. 2012) and tumor size (Wang *et al*. 2007). However; most of the RNA molecules reported to be differentially expressed in fibroids vs myometrium are microRNA (miRNA) species. LncRNAs form a specific group of non-coding RNAs (Woo & Kingston 2007) transcribed from ultraconserved intergenic regions and implicated in complex mechanisms of gene regulation such as epigenetic mechanisms (Mattick 2003, Costa 2008). Growing evidence for the implication of lncRNAs in tumorigenesis has been accumulating and, importantly, some of them have been shown to be significant predictors of tumor progression (Bussemakers *et al*. 1999, Ji *et al*. 2003, Yu *et al*. 2008, Gupta *et al*. 2010, Veltri 2014).

While several lines of evidence for the role of FH mutations in HLRCC have been reported, the exact mechanism of pathogenesis is still under study. The current paradigm invokes up-regulation of hypoxia-inducible factors (HIF) and hypoxia responsive genes in tumors with reduced expression of mitochondrial *FH* (Eng *et al*. 2003, Pollard *et al*. 2005, Ashrafian *et al*. 2010). However, the mechanism contributing to HIF activation (pseudo-hypoxic drive, increase in reactive oxygen species, defective apoptotic mechanism or anabolic drive resulting from accumulation of glycolytic intermediates) is still a debate. Our study failed to identify frequent FH mutations that would be consistent with the common nature of nonsyndromic UL but cannot exclude FH as a candidate target regulated by the associated lncRNA.

*RGS7* and *PLD5* are suspected adiposity genes (Aissani *et al*. 2006, Wang *et al*. 2011, Aissani 2014) that may indirectly affect tumor growth through changes in the hormonal milieu. For instance, by decreasing the serum level of SHBG, which may result in increased bioavailability of estrogen (Schwartz *et al*. 2000). The report of genetic linkage between this genomic region of chromosome 1q43 with the level of SHBG in the HERITAGE family study (Ukkola *et al*. 2002) may not be fortuitous. Nonetheless, co-localization of signals for several correlated traits and diseases (adiposity, UL, age-at-menopause, serum SHBG, predisposing for prostate cancer) to a genomic region overlapping the lncRNA locus may reflect complex linkage disequilibrium between 1q43 loci with significant effect size, pleiotropy or coordinated gene expression (Aissani 2014).

We have modeled tumor size as an ordinal variable with a three-level (case-only) or a four-level outcome variable that included UL-free controls. The design that includes the controls is important to test because misclassification of cases with small, ultrasound-undetectable tumors (<0.5 cm of diameter) as controls can be substantial (Baird *et al*. 2003), especially in cross-sectional studies. In contrast to the risk SNP rs2341938, rs2654879 and rs316912 SNPs associated only with tumor size and not with both outcomes. A possible explanation would be that the former SNP tags a null mutation while the latter tag variants affecting the level of gene expression.

Disentangling the genetic correlates of syndromic vs nonsyndromic UL in the studied genomic region on chromosome 1q43 is an important future undertaking to improve our understanding not only of UL pathogenesis but also of the genetic mechanisms underlying syndromic forms (rare Mendelian disease) and nonsyndromic (common diseases) counterparts of diseases in general.

## Supplementary data

This is linked to the online version of the paper at http://dx.doi.org/10.1530/ERC-15-0208.

## Patient consent

All participants provided written informed consent, including permission to perform genetic analysis.

## Ethics approval

All studies involved in this research received institutional review board approvals.

## Author contribution statement

All listed authors have substantially contributed to the work. B Aissani, K Zhang and H W Wiener conceived and designed the experiments. A R Mensenkamp and F H Menko investigated HLRCC patients and contributed samples. H W Wiener analyzed the data; B Aissani and K Zhang provided analytical support. All authors contributed to the interpretation of the results. B Zhang drafted the manuscript and all authors reviewed/edited manuscript. All authors approved the final manuscript.

## Figures and Tables

**Table 1:** Mutational analysis of the fumarate hydratase gene in syndromic and nonsyndromic uterine leiomyoma

**ID**	**Study**	**Race**	**Sex**	**Affection status**^a^	**HLRCC**			**SNP**	**Position** (bp)	**Allele**		**DNA change**	**Protein change**	**MAF**
UL	CL	RCC	Ref	Alt
3633	HLRCC	EUR	2	1	1	1	0		241 665 769	C	A	c.1210G>T	p.Gln404X	
3738	HLRCC	EUR	2	1	1	1	0		241 665 769	C	A	c.1210G>T	p.Gln404X	
6229	HLRCC	EUR	2	1	1	1	0		241 665 790	C	T	c.1189G>A	p.Gly397Arg	
7551	HLRCC	EUR	2	1	1	1	0		241 665 835	T	C	c.1144A>G	p.Met382Val	
4760	HLRCC	EUR	2	1	1	1	0		241 667 448	A	C	c.1002T>G	p.Ser334Arg	
1028	HLRCC	EUR	2	0	0	1	0	rs398123168	241 667 498	G	A	c.952C>T	p.His318Tyr	
2542	HLRCC	EUR	2	1	1	1	0		241 669 383	C	T	c.824G>A	p.Gly275Glu	
4856	HLRCC	EUR	2	1	1	1	0		241 669 383	C	T	c.824G>A	p.Gly275Glu	
3975	HLRCC	EUR	2	1	1	1	0	rs121913123	241 671 943	C	T	c.698G>A	p.Arg233His	
10755	HLRCC	EUR	2	1	1	1	0	rs121913123	241 671 943	C	T	c.698G>A	p.Arg233His	
796	NIEHS-UFS	AA	2	1	NA	NA	NA		241 682 968	C	A	c.55G>T	p.Ala19Ser	0.0054
629	NIEHS-UFS	AA	2	1	NA	NA	NA	rs201887750	241 682 970	G	A	c.53C>T	p.Pro18Leu	0.011
1065	NIEHS-UFS	AA	2	0	NA	NA	NA	rs201887750	241 682 970	G	A	c.53C>T	p.Pro18Leu	0.011

HLRCC, hereditary leiomyomatosis and renal cell cancer; NIEHS-UFS, National Institute of Environmental Health Science uterine fibroid study; EUR, European; AA, African American; UL, uterine leiomyoma; CL, cutaneous and leiomyoma; RCC, renal cell cancer; SNP, single nucleotide polymorphism; ref, reference allele; alt, alternative allele; MAF, minor allele frequency. All positions are according to the human genome assembly 19 (GRCh37/hg19).

a Syndromic or non-syndromic uterine leiomyoma (1=affected; 0=unaffected).

**Table 2:** List of chromosome 1q43 single nucleotide polymorphisms associated with risk or size of uterine leiomyoma in the NIEHS uterine fibroid study

**SNP**	**Inter SNP distance** (bp)	**Minor allele frequency**		**Gene/function**	**Location**	**eQTL-associated genes, *P***	**Pooled sample analysis, *P***			**Meta-analysis**			
		EA	AA				Risk	Tumor size		Risk		Tumor size	
Four-level	Three-level	*P*	*P*-het	*P*	*P*-het
rs4660080A>G	–	0.44	0.49	*RGS7*-lnc-RNA	Intergenic	–	2.1×10^−4^	9.4×10^−5^	1.9×10^−2^	–	0.00	–	0.03
**rs2341938**G>A	3958	0.44	0.46	*RGS7*-lnc-RNA	Intergenic	NA	1.6×10^−4^	5.5×10^−5^	1.9×10^−2^	–	0.00	–	0.03
**rs78220092**C>A	1829	0.006	0.14	*RGS7*-lnc-RNA	Intergenic	NA	–	–	–	5.4×10^−5^	0.99	–	0.99
rs1891129C>T	38 107	0.46	0.49	lnc-RNA	Upstream	*FH*, 0.001	1.7×10^−2^	2.8×10^−3^	3.7×10^−2^	–	0.02	–	0.04
rs28627534G>A	569 850	0.46	0.46	*MAP1LC3C*	Downstream	NA	–	–	–	–	0.49	1.8×10^−3^	0.43
rs316912T>C	278 911	0.00	0.03	*PLD5*	Intron	NA	–	2.7×10^−2^	4.8×10^−4^	–	1.00	6.0×10^−4^	1.00
**rs2654879**T>C	165 158	0.45	0.31	*PLD5*	Intron	–	–	2.0×10^−3^	1.7×10^−4^	–	0.49	1.8×10^−3^	0.43
rs6429360G>A	53 876	0.05	0.41	*PLD5*	Intron	–	4.1×10^−4^	8.9×10^−3^	–	–	0.00	–	0.60

SNP, single nucleotide polymorphism; EA, European American; AA, African American; eQTL, expression quantitative trait locus; *P*-het, *P* value for the test of heterogeneity; lnc-RNA, large intergenic non-coding RNA; *RGS7*, regulator of G-protein signaling 7; *FH*, fumarate hydratase; *MAP1LC3C*, microtubule-associated protein 1 light chain 3 gamma; *PLD5*, phospholipase D family, member 5; –, not significant at *P*=0.05 or that the assumption for proportional odds was not met; NA, non-apply (no information available). SNPs shown in bold are those that reached nearly or statistically significant association (Bonferroni-adjusted *P*<0.05) with either risk or size of UL and in either race-stratified analyses, combined analyses or meta-analyses.
